# Endometrial apoptosis and neutrophil infiltration during menstruation exhibits spatial and temporal dynamics that are recapitulated in a mouse model

**DOI:** 10.1038/s41598-017-17565-x

**Published:** 2017-12-12

**Authors:** Gregory M. Armstrong, Jacqueline A. Maybin, Alison A. Murray, Moira Nicol, Catherine Walker, Philippa T. K. Saunders, Adriano G. Rossi, Hilary O. D. Critchley

**Affiliations:** 1 MRC Centre for Reproductive Health (CRH), University of Edinburgh, The Queen’s Medical Research Institute (QMRI), 47 Little France Crescent, Edinburgh, EH16 4TJ UK; 2 MRC Centre for Inflammation Research (CIR), University of Edinburgh, The Queen’s Medical Research Institute (QMRI), 47 Little France Crescent, Edinburgh, EH16 4TJ UK

## Abstract

Menstruation is characterised by synchronous shedding and restoration of tissue integrity. An *in vivo* model of menstruation is required to investigate mechanisms responsible for regulation of menstrual physiology and to investigate common pathologies such as heavy menstrual bleeding (HMB). We hypothesised that our mouse model of simulated menstruation would recapitulate the spatial and temporal changes in the inflammatory microenvironment of human menses. Three regulatory events were investigated: cell death (apoptosis), neutrophil influx and cytokine/chemokine expression. Well-characterised endometrial tissues from women were compared with uteri from a mouse model (tissue recovered 0, 4, 8, 24 and 48 h after removal of a progesterone-secreting pellet). Immunohistochemistry for cleaved caspase-3 (CC3) revealed significantly increased staining in human endometrium from late secretory and menstrual phases. In mice, CC3 was significantly increased at 8 and 24 h post-progesterone-withdrawal. Elastase^+^ human neutrophils were maximal during menstruation; Ly6G^+^ mouse neutrophils were maximal at 24 h. Human endometrial and mouse uterine cytokine/chemokine mRNA concentrations were significantly increased during menstrual phase and 24 h post-progesterone-withdrawal respectively. Data from dated human samples revealed time-dependent changes in endometrial apoptosis preceding neutrophil influx and cytokine/chemokine induction during active menstruation. These dynamic changes were recapitulated in the mouse model of menstruation, validating its use in menstrual research.

## Introduction

Menstruation is an inflammatory process characterised by breakdown and shedding of the endometrium, bleeding and recruitment of migratory leucocyte populations. Resolution of inflammation at and following menstruation is critical to limiting tissue damage and to efficient repair of the endometrium. Apoptosis and clearance of apoptotic cells are critical to the successful resolution of inflammation elsewhere in the body^1^, however the relative timing and extent of apoptosis with respect to inflammation and its resolution in the endometrium have yet to be well characterised.

The endometrium consists of a simple columnar epithelium overlying a multicellular stroma. The stroma comprises connective tissue with fibroblast-like stromal cells and contains a number of tubular glands contiguous with the luminal surface, spiral arteries and fluctuating populations of various recruited leucocytes.

Over the course of the menstrual cycle, the human uterus is exposed to an environment of cyclically expressed ovarian sex steroids which are crucial to the regulation of growth and differentiation of the endometrium^2,3^. Principal amongst these sex steroids are 17β-oestradiol (E_2_) and progesterone (P_4_), concentrations of which fluctuate in a well-characterised manner through the menstrual cycle.

The rapid decline in ovarian-derived progesterone that occurs when the corpus luteum involutes during a non-pregnant cycle triggers changes in endometrial function which culminate in the breakdown and piecemeal shedding of the upper, functional layer of the endometrium during menstruation. Leading up to menstruation, a number of histological changes in the endometrium are observed: tissue oedema^4^, extensive recruitment of circulating leucocytes, breakdown of the basal lamina supporting endothelial cells, and augmented blood vessel permeability and fragility^2,5^. These histological changes are further accompanied by molecular events, such as the focal activation of matrix metalloproteinases (MMPs) in regions of menstrual lysis^6,7^, increased cyclooxygenase-2 (COX-2)^8^ and a consequent increase in prostaglandins^9^. The similarities of these features to those of classical inflammation formed the basis for the first hypothesis of menstruation as an inflammatory event^4^.

Amongst the leucocytes to which the human endometrium is host through the menstrual cycle, neutrophil granulocytes are reported to be recruited in substantial numbers prior to menstruation^10^ – coincident with declining progesterone concentrations. Neutrophils have been estimated to comprise between 6–15% of the total endometrial cell numbers at this time^11^, and have been suggested to play an important role in not only the destruction of endometrial tissue at menstruation, but also in its concomitant repair^12^.

Apoptosis is a form of programmed cell death in which cells condense and fragment their nuclear material, condense their cytoplasmic material, and then release their contents in membrane-bound apoptotic bodies^13^. Cells are induced to undergo apoptosis through either extrinsic or intrinsic pathways, both of which converge on the cleavage of inactive pro-caspase-3 to active, cleaved caspase-3, an ‘executioner’ cysteine-aspartic acid protease (caspase) whose activation irreversibly initiates the cascade of apoptotic events^14^. Extrinsic apoptotic pathways lead to pro-caspase-3 cleavage by the ‘initiator’ caspase-8^15^, while intrinsic apoptotic pathways lead to pro-caspase-3 cleavage by the initiator caspase-9^16^.

Clearance of apoptotic cells by resident phagocytes represents a critical juncture in the transition from inflammation to resolution, acting both to deplete inflammatory cells from the site and to skew phagocytes to an anti-inflammatory phenotype^1,17^. In most acute inflammatory contexts, short-lived neutrophils represent the major infiltrating leucocyte constituent, and are therefore among the more abundant apoptotic cells encountered by professional phagocytes in the resolving inflammatory environment.

**Figure 1:**
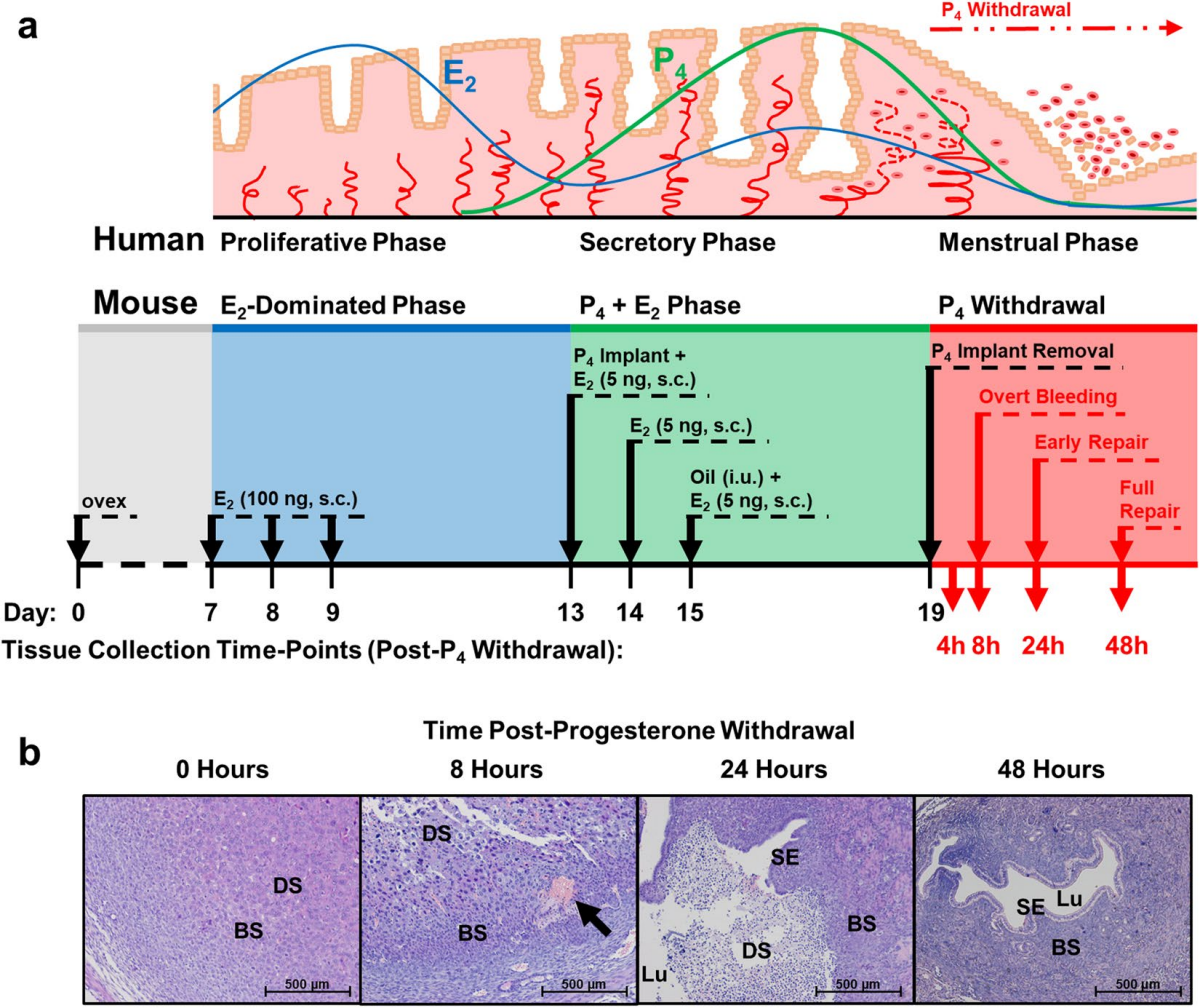
Schematic comparison of human menstrual cycle (top) and simulated menstruation protocol in an ovariectomised mouse model (bottom). (**a**) Upper panel depicts a schematic of the endometrium and its changes through the menstrual cycle consequent upon circulating concentrations of oestradiol (E_2_) and progesterone (P_4_). Lower panel depicts a schematic of the simulated menses protocol in the ovariectomised mouse model and the time-points at which tissues were collected. ovex = ovariectomy, s.c. = subcutaneous, i.u. = intrauterine. (**b**) Representative H&E photomicrographs of mouse endometrial tissue sections through decidualisation (0 h; stromal decidualisation, but no tissue breakdown), overt menses (8 h; loss of stromal compartment structural integrity), early repair (24 h; early re-epithelialisation and dissociation of necrotic tissue from basal endometrium) and full repair (48 h; stromal restoration and complete re-epithelialisation) following withdrawal of progesterone. Black arrow indicates active bleeding at 8 h, which can be seen both micro- and macroscopically. BS = basal stroma, DS = decidualised stroma, Lu = lumen, SE = surface epithelium.

Menstruation only occurs in mammals whose endometria spontaneously decidualise prior to implantation, such as humans and Old World primates. Mice, along with other mammalian species whose endometria do not undergo spontaneous decidualisation^18^, do not ordinarily menstruate. Nevertheless, menstruation can be simulated in mice, according to a well-established protocol^19,20^: ovariectomised mice are administered oestradiol injections and a subcutaneously implanted progesterone-releasing implant to mimic human endocrine regulation, after which the endometrium is stimulated to undergo decidualisation via the intrauterine injection of oil. Removal of the progesterone implant (mimicking regression of the corpus luteum in humans) triggers menstrual-like molecular and histological changes in the endometrium (Fig. 1).

Herein we detail the onset of apoptosis during the peri-menstrual phase (ovarian luteo-follicular transition) in carefully categorised human endometrium and in the mouse model of simulated menstruation. In addition, we identify the timing of neutrophil influx in both human and mouse endometrium. Finally, we reveal increased leucocyte chemokines and inflammatory mediators in human endometrium and the recapitulation in the mouse endometrium during ‘simulated’ menses.

## Results

### Human endometrial glands undergo widespread apoptosis in the late secretory phase, preceding menstrual shedding

**Figure 2:**
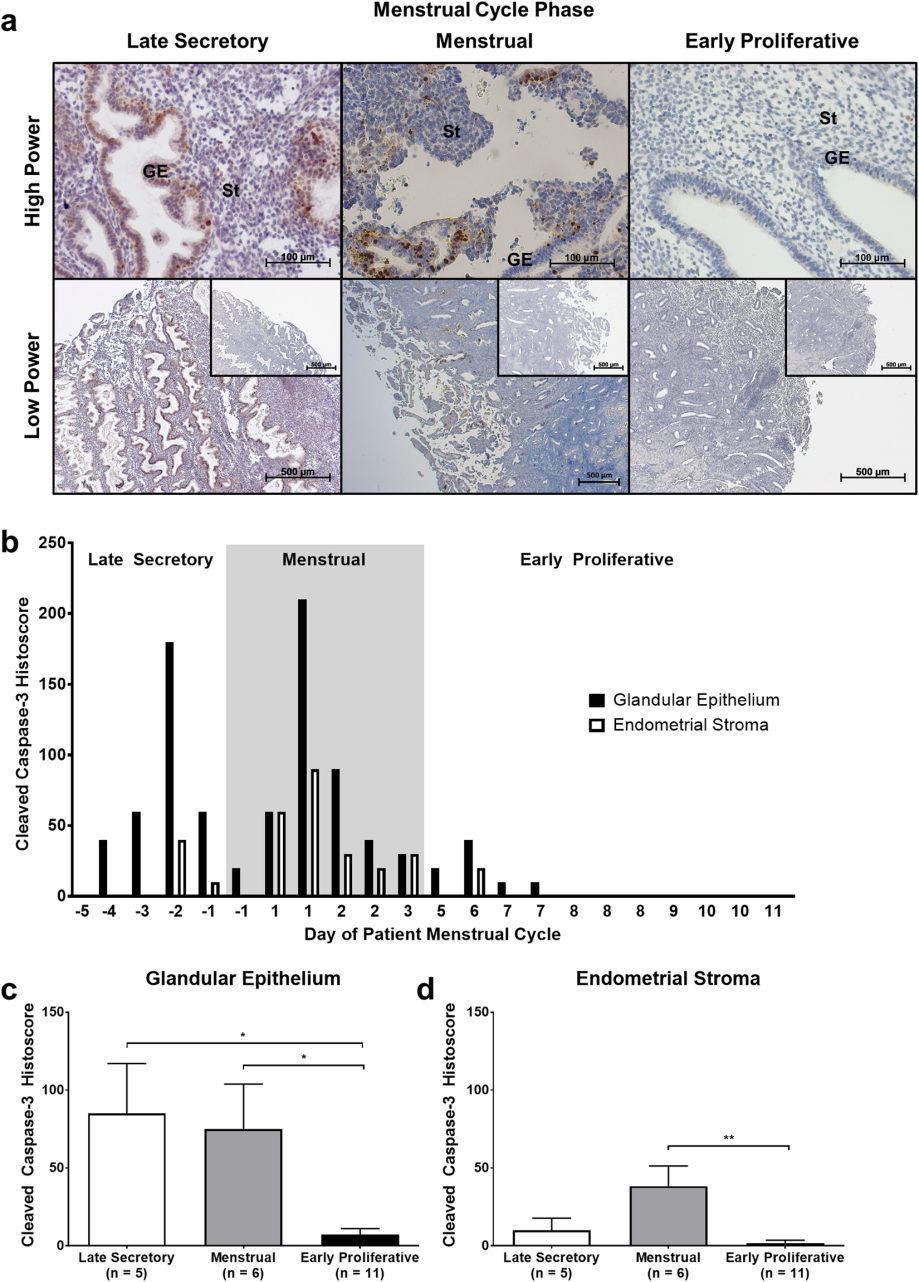
Apoptosis precedes menstrual shedding in the human endometrium. Expression of cleaved caspase-3 was analysed by immunohistochemistry and semi-quantitative histoscoring. (**a**) Representative photomicrographs of endometrial tissue sections at high power and low power with isotype control insets. GE = glandular epithelium, St = stroma. (**b**) Cleaved caspase-3 (CC3) expression in the endometrial glands and stroma plotted against day of patient menstrual cycle. Each x-value represents data from one individual patient sample, therefore some cycle days are repeated. Dark grey box indicates the period of menstruation. (**c**, **d**) CC3 expression in endometrial glands and stroma grouped by menstrual cycle phase. Results are presented as mean ± SEM, with results consistent across three technical replicates. Significance determined by 1-way ANOVA and Tukey’s multiple comparisons test. *p < 0.05, **p < 0.01.

Post-hysterectomy, full-thickness endometrial biopsies (from luminal endometrium to the endometrial/myometrial junction) were immunostained for the apoptotic marker, cleaved caspase-3 (CC3; Fig. 2a,b), to investigate the temporal and spatial organisation of apoptosis in the endometrium during the ‘peri-menstrual phase’. Endometrial biopsies were selected therefore to span from late secretory phase (wherein the concentration of progesterone is high and the concentration of oestradiol low) through to early proliferative phase (wherein the concentration of progesterone is low and the concentration of oestradiol high).

Cleaved capsase-3 was strongly stained in the glandular epithelium from late secretory phase, preceding overt menstrual bleeding. Cleaved caspase-3 staining continued through into the menstrual phase before significantly decreasing by the early proliferative phase (Fig. 2c). Cleaved caspase-3 staining was also detected in the endometrial stroma, but much less intensely than in the glandular epithelium. Low level stromal staining persisted during the menstrual phase, particularly localised to the functional layer (Fig. 2).

These results suggest a coordinated induction of apoptosis in the human endometrium as progesterone concentrations decline (though in advance of menstruation proper), with apoptotic cells apparent first in the glandular epithelium before appearing in the stromal compartment.

### Apoptosis is detected in the mouse endometrium 8 and 24 hours following progesterone withdrawal

**Figure 3:**
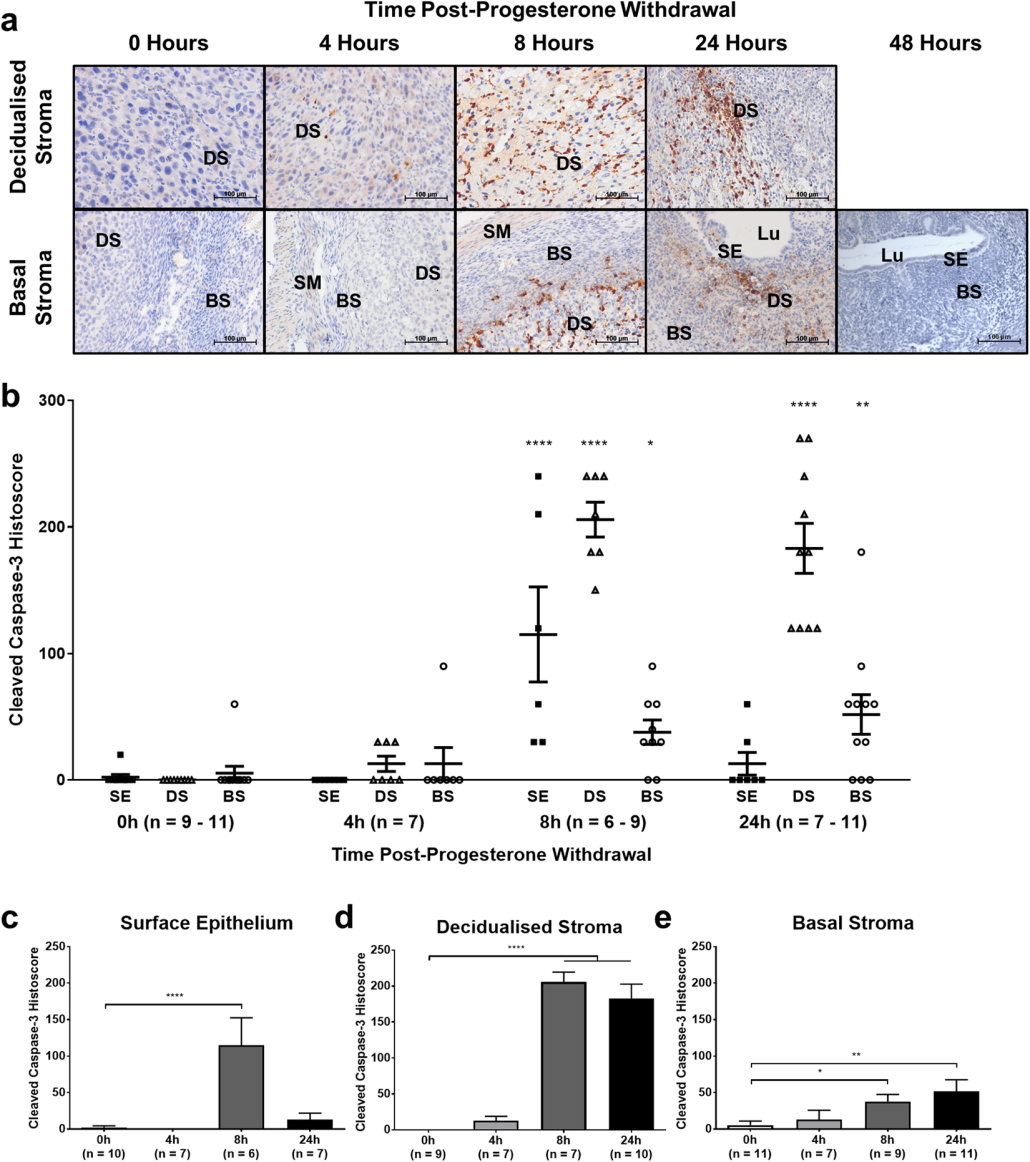
Apoptosis is significantly increased by 8 hours after progesterone withdrawal in the mouse endometrium. Expression of CC3 was analysed by immunohistochemistry and semi-quantitative histoscoring. (**a**) Representative photomicrographs of endometrial tissue sections at high power, centred on decidualised stroma and basal stroma. BS = basal stroma, DS = decidualised stroma, Lu = lumen, SE = surface epithelium, SM = smooth muscle. (**b**) CC3 expression in the surface epithelium (SE; black squares), decidualised stroma (DS; black triangles) and basal stroma (BS; black circles) plotted against time post-progesterone-withdrawal. (**c**–**e**) Expression in individual tissues plotted against time post-progesterone withdrawal. Results are presented as mean ± SEM. Significance determined by 1-way ANOVA and Dunnett’s multiple comparisons test (to 0 hours’ progesterone withdrawal). *p < 0.05, **p < 0.01, ****p < 0.0001.

A mouse model of simulated menstruation was employed to investigate whether the timing and extent of apoptosis observed in the human endometrium was simulated in the mouse endometrium following progesterone withdrawal. Immunostaining for cleaved caspase-3 in the mouse model revealed an induction of apoptosis preceding menstrual bleeding (Fig. 3a,b), consistent with observations in the human endometrium (Fig. 2). Cleaved caspase-3 immunostaining was heterogeneous at 8 and 24 hours following progesterone withdrawal, with strongest expression in the decidualised stroma and adjacent areas of basal stroma. By 48 hours following progesterone withdrawal, full endometrial repair had been effected, there was no longer any decidualised stroma, and no cleaved caspase-3 immunostaining was seen in either the surface epithelium or basal stroma.

Stromal decidualisation in the mouse endometrium makes assessment of glandular epithelium unreliable, therefore the decidualised mass and surface epithelium were assessed. Cleaved caspase-3 peaked in the surface epithelium and underlying stroma of the mouse endometrium 8 hours after progesterone withdrawal. Thereafter, staining decreased substantially in the surface epithelium by 24 hours (Fig. 3c) but remained significantly increased in the decidualised stroma (Fig. 3d). In tissues lying basal to the decidualised endometrial tissue (‘basal’ stroma), cleaved caspase-3 staining increased significantly by 8 hours after progesterone withdrawal and further still by 24 hours, but had a lower histoscore than the decidualised stroma compartment (Fig. 3e).

Apoptosis was rapidly induced in the progesterone-withdrawn, artificially decidualised mouse endometrium, recapitulating the phenomenon seen in the human endometrium at menses.

### Neutrophil abundance increases dramatically in the menstrual phase of the human endometrium

**Figure 4:**
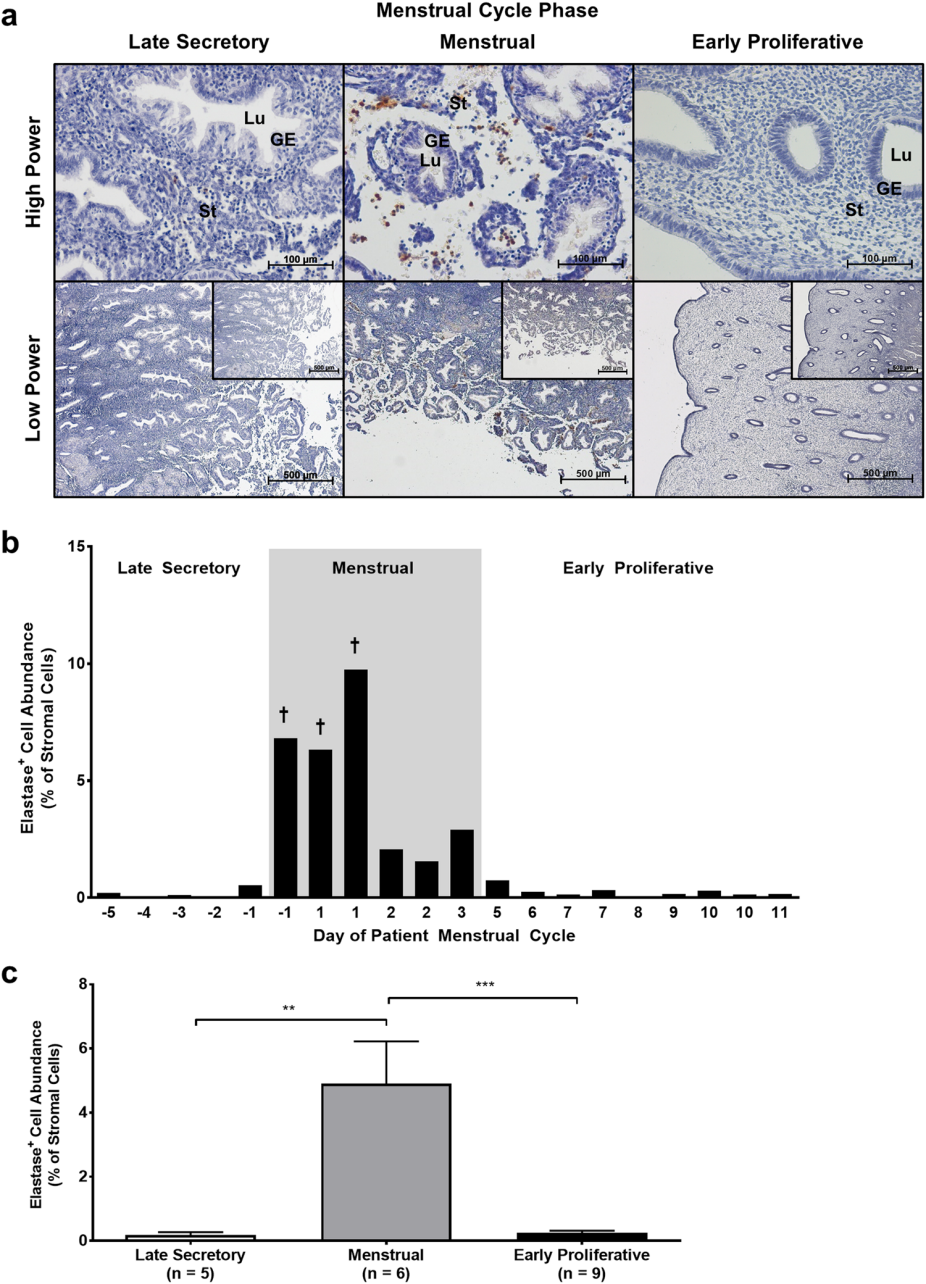
Neutrophil abundance peaks at the onset of menstruation in the human endometrium. Neutrophil abundance was analysed by immunohistochemistry and ImageJ-assisted stereology. (**a**) Representative photomicrographs of endometrial tissue sections at high power and low power with isotype control insets. GE = glandular epithelium, Lu = lumen, St = stroma. (**b**) Percentage of endometrial stroma comprised by elastase-immunopositive cells (normalised to total cell nuclei) plotted against day of patient menstrual cycle. Each x-value represents data from one individual patient sample, therefore some cycle days are repeated. † symbol indicates %SE (standard error) < 5. Dark grey box indicates the period of menstruation. (**c**) Stromal neutrophil abundance grouped by menstrual cycle phase. Results are presented as mean ± SEM, with results consistent across three technical replicates. Significance determined by 1-way ANOVA and Tukey’s multiple comparisons test. **p < 0.01, ***p < 0.001.

Investigating changes in neutrophil abundance across the peri-menstrual phase of the menstrual cycle revealed a tightly temporally regulated influx of elastase-immunopositive cells (Fig. 4a–c).

Neutrophil populations were consistently sparse in late secretory phase endometrial samples, however numbers of neutrophils increased dramatically by early menstrual phase (Fig. 4b). This influx of neutrophils was short-lived, decreasing by late menstrual phase and further still by the early proliferative phase to nearing pre-menstrual levels. Mean endometrial neutrophil abundance was significantly greater in the menstrual phase endometrium than in either late secretory or early proliferative phases (Fig. 4c).

These results indicate that neutrophil populations in the endometrium are tightly controlled, with influx being both rapidly induced and short-lived.

### Neutrophil populations increase significantly in the mouse endometrium after progesterone withdrawal

**Figure 5:**
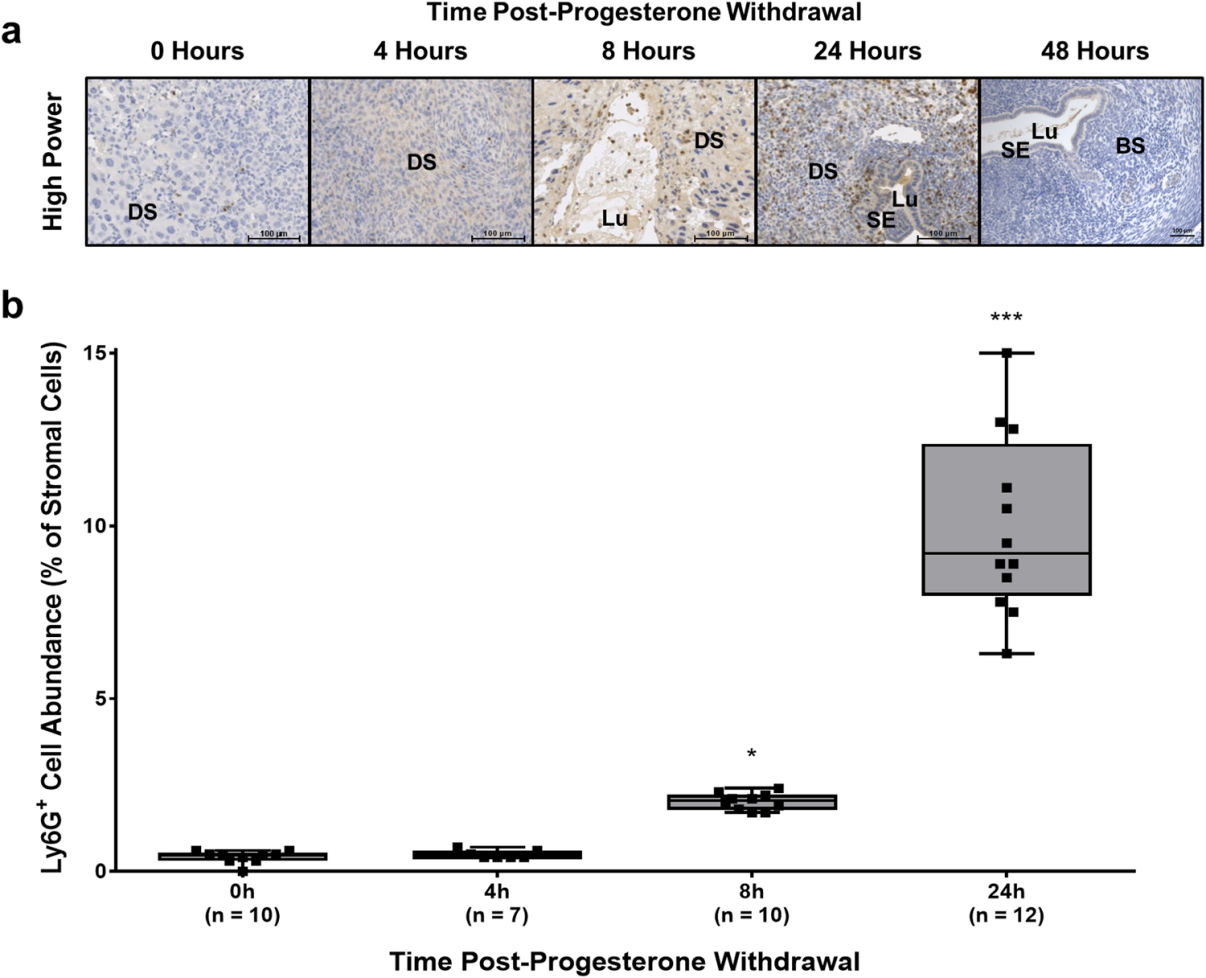
Neutrophil abundance is increased by 8 hours and peaks at 24 hours after progesterone withdrawal in the mouse endometrium. Neutrophil abundance was analysed by immunohistochemistry and ImageJ-assisted stereology. (**a**) Representative photomicrographs of endometrial tissue sections at high power. DS = decidualised stroma, Lu = lumen, SE = surface epithelium. (**b**) Percentage of endometrial stroma comprised by Ly6G-immunopositive cells (normalised to total cell nuclei) plotted against time post-progesterone-withdrawal. Results are presented as a boxplot constructed from second and third quartiles, with whiskers extending to minimum and maximum values. Significance determined by 1-way ANOVA and Dunnett’s multiple comparisons test (to 0 hours’ progesterone withdrawal). *p < 0.05, ***p < 0.001.

The mouse endometrium was similarly subject to significant increases in neutrophil abundance following the withdrawal of progesterone, as evidenced by immunostaining for the neutrophil marker, Ly6G (Fig. 5a,b).

Ly6G-immunopositive cell abundance was increased significantly by 8 hours following progesterone withdrawal (Fig. 5b), at which time neutrophils were principally localised to within the decidualised endometrial stroma (Fig. 5a). By 24 hours after progesterone withdrawal, Ly6G-immunopositive cell abundance was further increased (Fig. 5b), with neutrophils increasingly frequently localised to beneath the newly epithelialising luminal surfaces (Fig. 5a) – in contrast, this sub-luminal localisation of neutrophils was not observed in the human endometrium. By 48 hours after progesterone withdrawal, when full endometrial repair has been effected, no Ly6G-immunopositive cells were observed in the mouse endometrium.

Thus neutrophil abundance in the mouse endometrium was tightly regulated, again closely simulating the events in the human endometrium at menstruation.

### Leucocyte chemokines and inflammatory cytokines are maximal in the menstrual phase of the human endometrium

**Figure 6:**
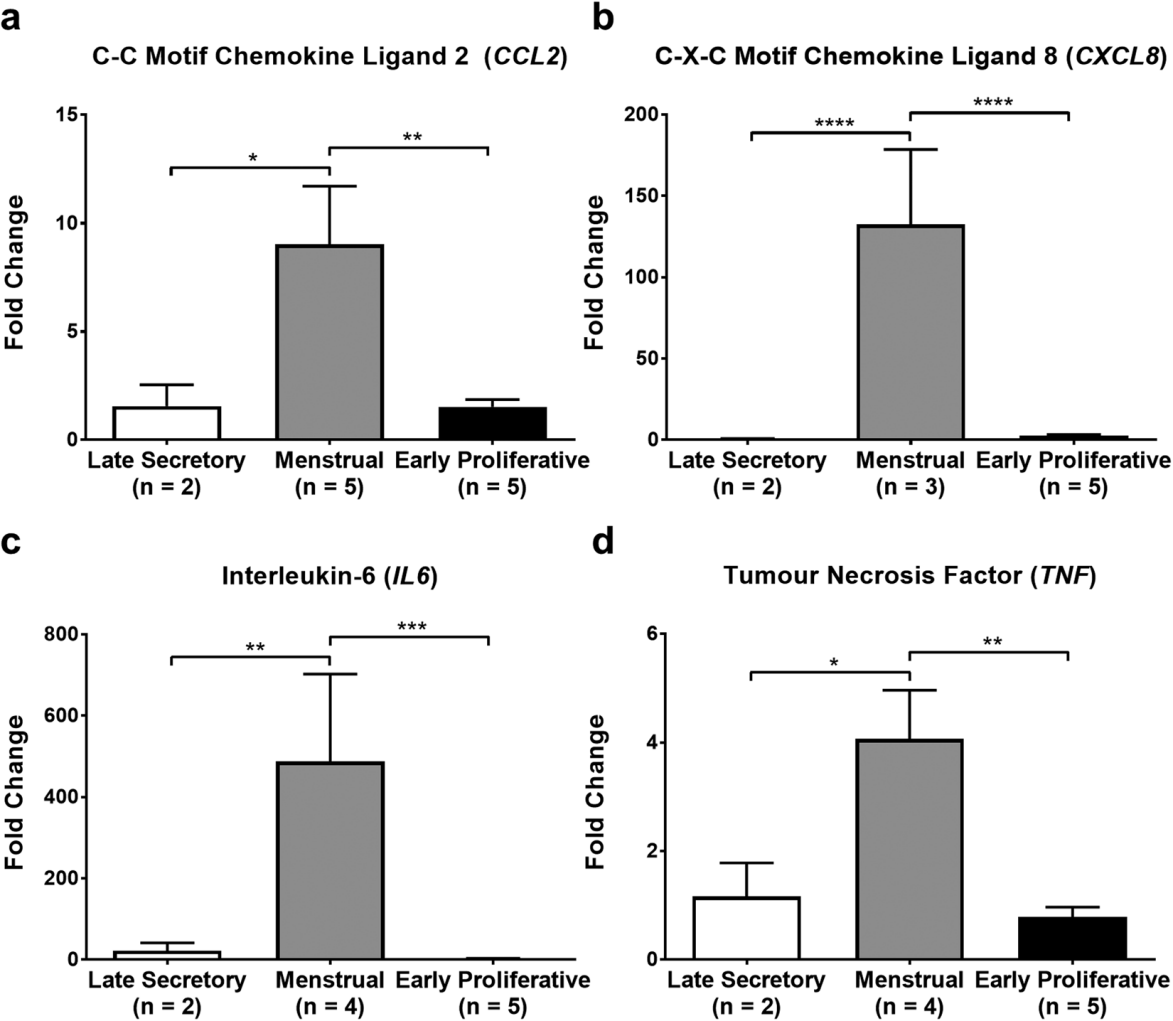
Expression of inflammatory cytokines and chemokines peaks during menstruation in the human endometrium. mRNA levels of (**a**) *CCL2*, (**b**) *CXCL8*, (**c**) *IL6* and (**d**) *TNF* determined by RT-qPCR and normalised to *ATP5B* mRNA levels. Data were analysed by ΔΔC_q_ method. Results are presented as mean ± SEM, with results consistent across three technical replicates. Significance determined by 1-way ANOVA and Tukey’s multiple comparisons test. *p < 0.05, **p < 0.01, ***p < 0.001, ****p < 0.0001.

mRNA concentrations of the chemokines CCL2 and CXCL8, and of the cytokines IL-6 and TNF, were raised significantly in menstrual phase endometrium compared to both late secretory and early proliferative phase endometrium (Fig. 6a–d).

These data demonstrate that endometrial inflammatory mediators are associated with neutrophil recruitment observed in the human endometrium following progesterone withdrawal.

### Leucocyte chemokines and inflammatory cytokines increase in the mouse endometrium following progesterone withdrawal

**Figure 7:**
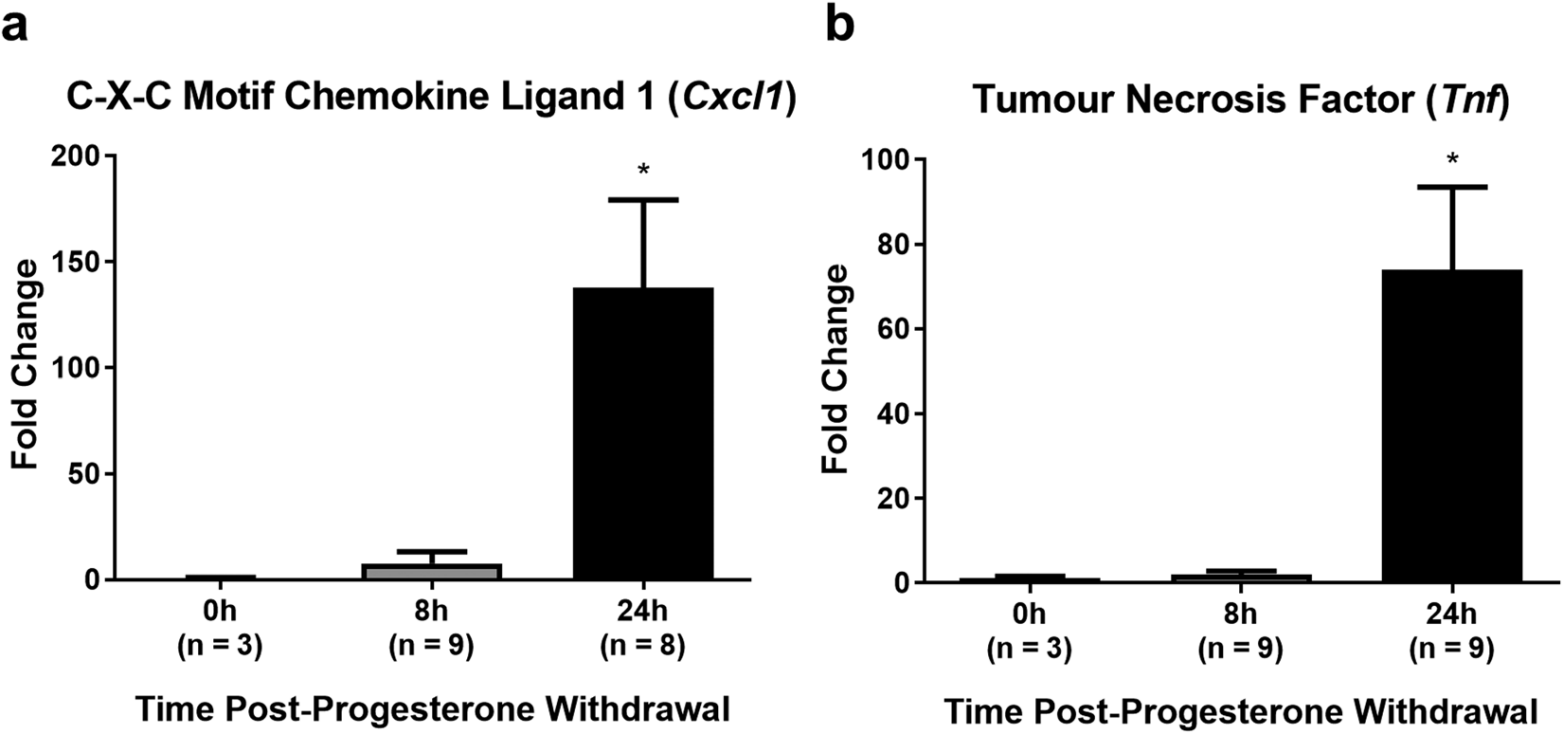
Expression of neutrophil chemokine CXCL1 and inflammatory cytokine TNF peaks at 24 hours after progesterone withdrawal in the mouse endometrium. mRNA levels of (**a**) *Cxcl1* and (**b**) *Tnf* determined by RT-qPCR and normalised to *Actb* mRNA levels. Data were analysed by ΔΔC_q_ method. Results are presented as mean ± SEM, with results consistent across three technical replicates. Significance determined by 1-way ANOVA and Dunnett’s multiple comparisons test (to 0 hours’ progesterone withdrawal). *p < 0.05.

Transcript levels of the chemokine CXCL1 (mouse homologue of human CXCL8) and of the cytokine TNF were significantly elevated in the mouse uterus at 24 hours following progesterone withdrawal (Fig. 7a,b) compared to tissue exposed to progesterone (0 hours).

These data indicate an increase in endometrial inflammatory mediators following progesterone withdrawal in the mouse model, coincident with neutrophil recruitment observed in the mouse endometrium and mimicking observations made in the human endometrium at menstruation.

## Discussion

In this report, the cellular and histological events occurring in the mouse endometrium during simulated menstruation were demonstrated to recapitulate the events that occur in the human endometrium at menstruation, specifically, apoptosis preceding cytokine/chemokine expression and extensive neutrophil influx.

We have shown that apoptosis occurs in the endometrium before both menstrual inflammation and overt shedding, and persists beyond the cessation of menstruation. Apoptosis may be among the earliest histological changes detected in the human endometrium leading up to menstruation. Likewise, in a mouse model of menstruation, endometrial apoptosis is rapidly detectable 4 hours after withdrawal of progesterone, and by 8 hours, apoptosis is widespread throughout the endometrium. As in the human endometrium, apoptosis precedes overt menstrual shedding and persists into post-menstrual endometrial repair. While apoptosis precedes inflammatory changes in the human and the mouse endometrium temporally, it is important to note that both apoptosis and inflammation are consequent upon the withdrawal of progesterone – no direct causal relationship is suggested between apoptosis and the subsequent inflammation.

The influx of neutrophils in the human endometrium was shown to occur dramatically at the onset of menstruation, after which neutrophil abundance decreased steadily through the menstrual phase. By the early proliferative phase of the menstrual cycle, neutrophils were all but absent from the human endometrium.

In the mouse model, endometrial neutrophil abundance was virtually negligible until 8 hours after the withdrawal of progesterone, whereupon the proportion of neutrophils in the stroma increased significantly. By 24 hours after progesterone withdrawal, neutrophils comprised approximately 10% of the endometrial stroma.

The human data presented in this report were plotted from individual patient samples, each meticulously characterised and selected to span the days preceding, during and following menstruation, *i*.*e*. during the luteo-follicular transition. The histological data were generated from ‘full-thickness’ endometrial biopsies retaining the myometrial-endometrial junction and uterine lumen architecture, thereby providing more reliable and complete measurements of apoptosis through the entirety of the endometrium than would otherwise have been achieved by other methods of endometrial sampling (*e*.*g*. ‘Pipelle’ tissue biopsies), where the spatial architecture may be lost. The RT-qPCR data were generated from endometrial biopsies collected from women with objectively verified ‘normal’ menstrual bleeding (*i*.*e*. < 80 mL per cycle)^21^, providing a tightly defined sample cohort.

Mean cleaved caspase-3 was significantly higher in the glands of the late-secretory-phase and menstrual-phase endometrium than in the glands of the proliferative-phase endometrium, and although less intense than in the glandular epithelium, was significantly higher in the stroma of the menstrual-phase endometrium than in the proliferative-phase endometrium. Cleaved caspase-3 in the luminal epithelium of the endometrium is likely to follow a similar course to that of the glandular epithelium, though reliable measurements proved difficult to obtain due to extensive luminal shedding in the menstrual phase. These data were therefore not shown.

Previous investigations of apoptosis in the endometrial glands have concerned themselves only with proliferative-phase samples^22^, wherein the authors sought to determine the extent of apoptosis by the broad agreement of terminal deoxynucleotidyl transferase (TdT)-mediated dUTP-biotin nick end labelling (TUNEL) staining with morphological apoptotic criteria^13^.

In the investigations of endometrial apoptosis undertaken herein, cleaved caspase-3 immunostaining was employed as a method of detecting apoptosis, in place of TUNEL staining. Whereas TUNEL staining is prone to false negatives (*e*.*g*. cells undergoing apoptosis which have not yet fragmented their chromatin^23^) and false positives (*e*.*g*. cells undergoing active gene transcription^24^, autolysis^25^, or exposed to certain tissue fixation and processing techniques^26^), cleaved caspase-3 immunostaining is not^27^.

Mean cleaved caspase-3 staining in the mouse endometrium was significantly increased in the luminal epithelium, decidualised and underlying basal stroma at 8 hours after progesterone withdrawal relative to 0 hours. By 24 hours after progesterone withdrawal, cleaved caspase-3 remained significantly increased in the decidualised and basal stroma relative to 0 hours, but not in the surface epithelium – likely due to the extensive re-epithelialisation occurring at this time, thereby reducing the proportion of epithelial cells that are apoptotic.

Previous studies investigating apoptosis in the progesterone-withdrawn mouse endometrium^19,28^ have not compared the localisation of apoptosis within the endometrium to specific tissue compartments as this report has. One of these studies^28^ effected the withdrawal of progesterone in the endometrium via the introduction of a progesterone receptor antagonist (mifepristone/RU486), effectively withdrawing progesterone pharmacologically. In contrast, withdrawal of progesterone was achieved in the current study via the removal of a progesterone-releasing implant – a method likely to better simulate the rapid regression of the corpus luteum prior to human menstruation.

In one of the few studies to date to have investigated neutrophil influx in the human endometrium in detail, neutrophils were found to comprise some 6–15% of the endometrial stroma on menstrual days 26–28 (which the authors designate ‘menses’)^11^. The data presented in this current report show a stromal neutrophil (elastase^+^) abundance of not more than 1% in samples from days 26–28 and not more than 1–10% of total stromal cells from women with active bleeding (days 1–3; mean ± SEM = 4.902 ± 1.321%). This slight difference in neutrophil abundance may be due to the inclusion in this report of data from “later” menstrual samples (days 2–3), when neutrophils are less abundant than menstrual onset (day 1). The use of ‘full-thickness’ endometrial samples (consistent for histological appearance, reported last menstrual period and serum oestradiol and progesterone concentrations at the time of endometrial sampling) in this report nevertheless permitted a detailed study where endometrial architecture from myometrial-endometrial junction to the upper functional layer-luminal surface was retained.

In mouse models, neutrophil abundance in the progesterone-withdrawn endometrium has also been investigated^29,30^, albeit not without some important limitations. Previous studies relied on two different antibodies to detect neutrophils: one specific for the Ly6B.2 antigen (clone 7/4)^29^, expressed not only by neutrophils, but also by inflammatory monocytes and a subset of activated macrophages^31^; and one specific for granulocyte receptor 1 (Gr-1; clone RB6-8C5)^30^. Gr-1 comprises the related antigens Ly6G (which is granulocyte-restricted) and Ly6C (which is expressed by neutrophils, dendritic cells, monocytes, macrophages and lymphocytes^32^), both of which are detected by anti-Gr-1 antibodies^33^. The quantification of neutrophils undertaken in this current report employed a Ly6G-specific antibody (clone 1A8), which demonstrates no cross-reactivity with the more broadly leucocyte-expressed Ly6C^34^. Recent work also supports the lack of cross-reactivity of this antibody with monocytes or monocyte-derived macrophages in the mouse uterus^35^.

Investigation into transcriptional changes in inflammatory chemokines and cytokines herein revealed statistically significant increases in CCL2 (MCP-1), CXCL8 (IL-8), IL-6 and TNF in the menstrual-phase human endometrium relative to that of the late secretory and early proliferative phases.

Previous work has reported endometrial CCL2 immunoreactivity as being significantly higher in late-secretory-phase tissues than in early-secretory-phase tissues, but did not find any significant differences between late-secretory-phase and menstrual-phase tissues and neither was *CCL2* transcriptional regulation specifically addressed^36^.

CXCL8 is a chemoattractant and activator of neutrophils, stimulating their adhesion to and extravasation through the endothelium as well as their degranulation within tissues^37^. As with CCL2, CXCL8 is reportedly increased in response to TNF alongside other pro-inflammatory stimuli^38^.

In the human endometrium, *CXCL8* mRNA concentration and protein has been described across the menstrual cycle^39^ in stromal cells^40^ and in blood vessel perivascular cells^41^, with the highest expression described in the menstrual phase^39^.

Endometrial transcription of *IL6* across the menstrual cycle has previously been reported, with peak transcription demonstrated in the late secretory phase^42^; these studies failed, however, to investigate transcription in menstrual-phase samples. Subsequent work by these same authors corroborated these transcriptional data with protein data, demonstrating peak IL-6 protein in the late secretory phase, which was localised to the endometrial glands and their secretions^43^. As before, however, menstrual-phase endometrial samples were not included in these experiments.

In the human endometrium, TNF has been described in stromal and epithelial cells^44^, wherein it has been suggested to play a role in menstrual shedding^45^. Levels of *TNF* mRNA are reportedly increased in the endometrium during the late secretory phase^42^, though as noted previously, no menstrual-phase samples were included in this sample cohort.

The findings of this report therefore substantially build upon existing literature regarding *CCL2* transcription in the endometrium across the menstrual cycle and corroborate previous findings regarding *CXCL8* ^39,41^, though are the first to do so in endometrial tissues carefully characterised for menstrual cycle staging and for ‘normal’ menstrual blood loss. Increased *IL6* and *TNF* transcription in the menstrual phase endometrium have not, to our knowledge, been previously described in the literature.

Transcriptional changes in *Tnf* (TNF) and in the neutrophil chemokine gene *Cxcl1* (KC; GRO-α) in the mouse endometrium investigated in this report revealed significant increases in the transcription of both genes at 24 hours following progesterone withdrawal.

Previous studies have demonstrated a significant increase in the transcription of *Tnf* at 24 hours following progesterone withdrawal^30^, which is corroborated by the data presented in this report. Increased *Tnf* at 24 hours is also in line with their finding that transcription of the prostanoid synthesis pathway gatekeeper enzyme *Ptgs2* (cyclooxgenase-2; COX-2) is increased at 24 hours, as TNF is a known inducer of *Ptgs2* transcription^46^.

CXCL1 is a mouse homologue of the human neutrophil chemokine CXCL8^47^, and has not previously been demonstrated in the mouse endometrium after withdrawal of progesterone. The transcription of another mouse neutrophil chemokine and CXCL8 homologue, *Cxcl2* (GRO-β), has however been demonstrated significantly upregulated at 24 hours following progesterone withdrawal^30^, which supports and complements the findings of this report.

That *Cxcl1* transcription was increased at 24 hours’ post-progesterone withdrawal is in keeping with the neutrophil abundance data presented herein, which showed substantial increases in neutrophil numbers at 24 hours. It moreover strengthens the argument that the infiltration of large numbers of neutrophils is a ‘deliberate’, coordinated response, and lends credence to the hypothesis that neutrophils are important to the breakdown and repair of the mouse endometrium following progesterone withdrawal^12^.

**Figure 8:**
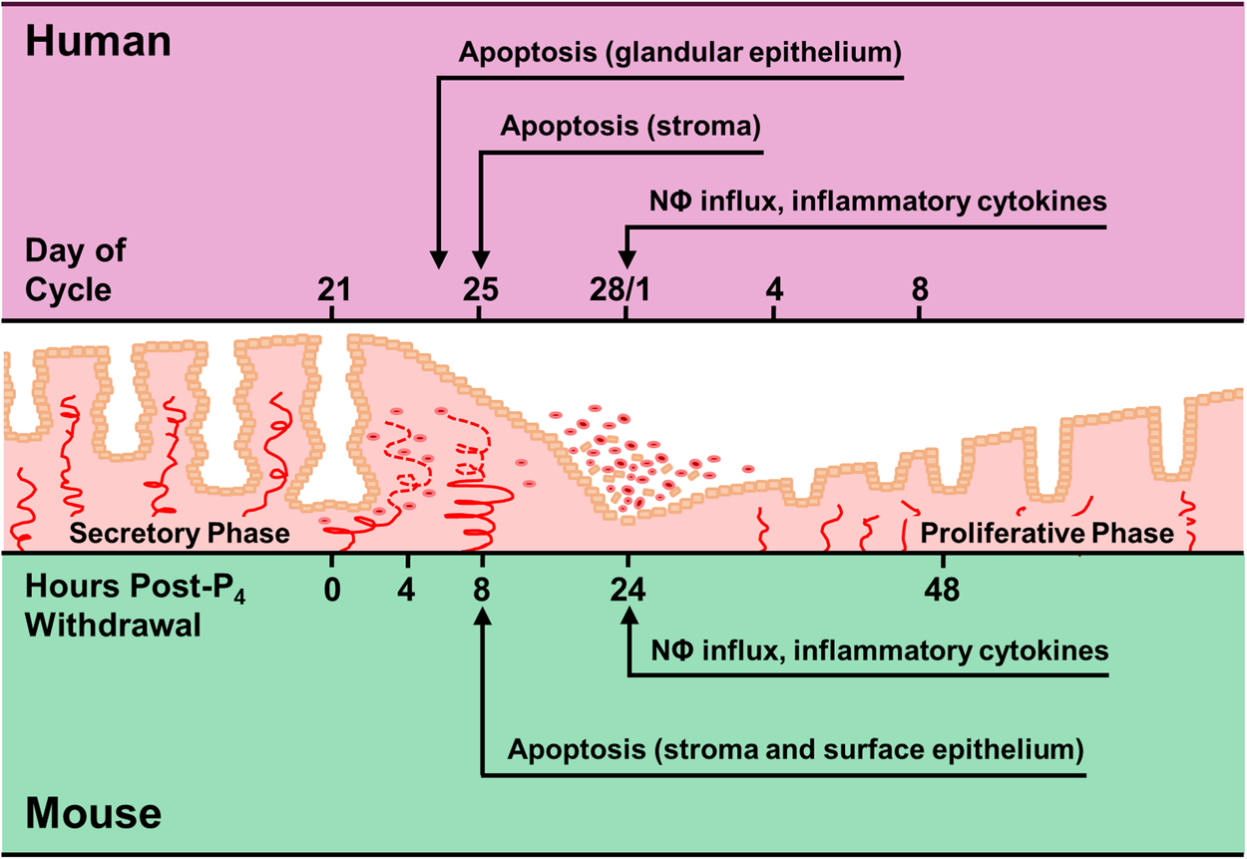
Summary of endometrial changes during menstruation in humans (top) and during simulated menstruation in an ovariectomised mouse model (bottom). Cellular and histological events occurring in the mouse endometrium during simulated menstruation recapitulate events that occur in the human endometrium at menstruation: apoptosis preceding extensive neutrophil influx and inflammatory cytokine expression. NΦ = neutrophils, P_4_ = progesterone.

Perhaps the most important finding of this report is the parallel that can be drawn between the observations made in the ‘peri-menstrual’ human endometrium and those made in the progesterone-withdrawn mouse endometrium in the ‘menses model’ (Fig. 8), strengthening the legitimacy of the model in recapitulating the events of human menstruation.

In the mouse, ‘menses’ occurs due to the removal of the progesterone-releasing Silastic implant; in the human, menses occurs due to the regression of the progesterone-producing corpus luteum. Nevertheless, features common to both the mouse and human endometrium are many: early endometrial apoptosis preceding overt menstrual bleeding and tissue destruction; substantial increases in infiltrating neutrophil granulocyte abundance; and increased transcription of inflammatory chemokines and cytokines (*Tnf*/*TNF*, *Cxcl1*/*CXCL8*).

In conclusion, these data from well-categorised human endometrium reveal progesterone withdrawal is followed by endometrial apoptosis, which precedes increased endometrial cytokine and chemokine presence and neutrophil influx during menstruation. These events are recapitulated in the mouse model of simulated menstruation, further validating its use as a tool for the investigation of endometrial physiology, pathology and menstrual disorders.

## Materials and Methods

### Human endometrium clinical samples

Twenty-two paraffin-embedded, post-hysterectomy endometrial tissue samples (luminal endometrium to endometrial/myometrial junction, *i*.*e*. ‘full-thickness’) for immunohistochemical (IHC) studies and, 11 endometrial ‘Pipelle’ biopsies (blind sampling technique from uterine cavity) for RT-qPCR studies were obtained from otherwise healthy women, whose menstrual cycle phases had been characterised by histological dating, last menstrual period and serum oestradiol/progesterone concentrations at the time of biopsy collection. Full exclusion criteria and menstrual cycle details are provided in Supplementary Table 1.

All participating patients provided informed, written consent. Samples were collected with Lothian Research Ethics Committee approval (1994/6/17; 05/S1103/14; 10/S1402/59). All procedures were conducted in accordance with relevant guidelines and regulations.

### Mouse endometrial samples and model of simulated menstruation

Menstruation was simulated in C57Bl/6 J mice (8–12 weeks, n = 31; Harlan Laboratories, Indianapolis, USA) according to an established protocol^19,20^ (Fig. 1). Mice were ovariectomised under isoflurane-induced anaesthesia with buprenorphine hydrochloride analgesic, and allowed to recover for a minimum of 7 days. Oestradiol was administered on 3 consecutive days (100 ng s.c.); 4 days later, a subcutaneous progesterone-releasing implant was inserted, and a lower dose of oestradiol was administered on 3 consecutive days (5 ng s.c.). On the last day of lower-dose oestradiol injections, endometrial decidualisation was stimulated by the injection of 18 μL of arachis oil into the lumen of one uterine horn (with the other horn acting as a non-decidualised control) using non-surgical embryo transfer (NSET) devices. Progesterone-withdrawal was achieved by the surgical removal of the progesterone-releasing implants under anaesthesia. Mice were sacrificed and endometrial tissues were collected at 0 (n = 9), 4 (n = 7), 8 (n = 6), 24 (n = 7) and 48 (n = 2) hours after progesterone withdrawal.

All mouse experiments were performed under a Home Office issued Personal Licence (I00B1A8F3) and Project Licence (60/4208). All mouse experiments were conducted in accordance with relevant guidelines and regulations.

### Immunohistochemistry and stereology

Immunohistochemical staining for cleaved caspase-3, neutrophil elastase and Ly6G was performed in 22 paraffin-embedded human endometrial tissues and 29 paraffin-embedded mouse endometrial tissue sections.

Neutral-buffered formalin-fixed, paraffin-embedded tissues were cut into 5-μm-thick sections, deparaffinised in xylene (10 minutes), rehydrated in decreasing concentrations of ethanol (100%, 95%, 80% and 70%; 20 seconds each) and subjected to heat-induced epitope retrieval in 0.01 M sodium citrate buffer (pH 6.0) at 127 °C (10 seconds). After cooling and washing in phosphate-buffered saline (PBS), tissues were incubated in 3% H_2_O_2_/methanol (10 minutes) to quench endogenous peroxidase activity, then incubated in non-immune block. Non-immune block consisted of serum from the species in which the secondary antibody was raised (goat or horse), diluted 1 in 5 in 5% w/v bovine serum albumin (BSA)/PBS.

Sections from tissues known to express high levels of proteins of interest were used as positive controls. For negative controls, equimolar concentrations of generic immunoglobulins from the same species in which the primary monoclonal antibodies were raised (or the immunoglobulin fraction of serum from non-immunised animals in which polyclonal antibodies were raised) were used in place of the primary antibodies.

Primary antibodies and their matched negative controls were as follows: anti-cleaved-caspase-3 (rabbit polyclonal antibody, 63 μg/mL used at 1:400; Cell Signalling Tech, Leiden, Netherlands) and rabbit ‘normal’ Ig (immunoglobin fraction of serum from non-immunised rabbits); anti-elastase (mouse monoclonal antibody, clone NP57, 0.22 μg/mL used at 1:500; Agilent Technologies, Stockport, UK) and mouse IgG1 κ; and anti-Ly6G (rat monoclonal antibody, clone 1A8, 0.5 μg/mL used at 1:1000; BioLegend, San Diego, USA) and rat IgG2a κ.

Tissues were incubated in humidified chambers overnight at 4 °C with the primary antibodies diluted to appropriate concentrations in 20% serum/BSA/PBS. After washing with PBS, tissues were then incubated at room temperature with peroxidase-labelled anti-mouse (horse), -rabbit (horse) or -rat (goat) secondary antibody (Anti-Mouse (MP7402); Anti-Rabbit (MP7401) and Anti-Rat (MP7444) and all were used at a dilution of 1:200 for 30 minutes; Impress kits from Vector Labs, Peterborough, UK), then washed again with PBS. Expression was visualised with 3,3′-diaminobenzidine (DAB) chromogen, and Harris’ haematoxylin counterstain.

A well-established semi-quantitative histoscoring system^48–50^, calculated by multiplying a staining intensity grade of 0–3 (0, no staining; 1, weak staining; 2, moderate staining; 3, strong staining) by an estimation of the percentage of immunopositive tissue within different cellular compartments (to the nearest 10%), was employed, yielding a histoscore of 0–300. Each data point represented an average taken from at least three different tissue section histoscores.

Cells of interest were counted by stereological microscopy: the area of tissue sections comprised by endometrium was manually delineated, and counting fields were selected automatically and randomly by the software. Immunopositive cells were counted from each field manually, while total cell numbers were counted with the assistance of a macro written for the open-source image manipulation software ImageJ1 to allow discrimination of cell nuclei on the basis of colour thresholds and minimum/maximum size criteria. Manual user intervention permitted the elimination of false positives and inclusion of false negatives obtained through this method. Immunopositive cell counts were normalised to total cell nuclei.

### RNA isolation and RT-qPCR

Total RNA was extracted from endometrial tissues using commercially available RNA extraction kits per the manufacturer’s instructions, with additional DNA digestion treatment of samples with DNase I. RNA concentrations were quantified and purities were verified by means of a spectrophotometer, after which RNA was reverse transcribed using commercially available kits (Invitrogen SuperScript Vilo cDNA synthesis kit (#11754-250; Life Technologies Ltd, Paisley, UK).

TaqMan-based RT-qPCR was performed using TaqMan Supermix (Thermo Fisher Scientific, Perth, UK), with three technical replicates. The data were normalised to internal housekeeping controls (*ATP5B*, human endometrium; *Actb*, mouse endometrium) which were validated prior to conducting the experiments. Data were analysed and processed with Sequence Detection System v2.3 software (Applied Biosystems, Foster City, USA).

### Statistical analyses

Standard errors for cell counting were calculated according to the equation, ${\rm{SE}}=\sqrt{\frac{{{\rm{P}}}_{pi}(100-{P}_{pi})}{{{\rm{P}}}_{T}}}$ where *P*_*pi*_ represents the percentage of points occupied by cells of interest and *P*_*T*_ represents the total number of points occupied by all cells. Percentage standard error was then calculated according to $\% \,SE=100 \% (\frac{SE}{Ppi})$.

All other statistical analyses were performed with GraphPad PRISM software (v6.0; GraphPad Software Inc., San Diego, CA). Data were analysed using 1-way ANOVA methods with Tukey’s or Dunnett’s multiple comparisons tests, and represented as the means ± SEM, where *p* < 0.05 was considered significant.

### Data Availability

The datasets generated and analysed during this study are included in this published article (and its Supplementary Information files), and are available from the corresponding author on reasonable request.

## Electronic supplementary material

Supplementary Information
