# Supplementary material for: Endometrial apoptosis and neutrophil infiltration during menstruation exhibits spatial and temporal dynamics that are recapitulated in a mouse model
Source: Sci Rep. 2017 Dec 12;7:17416. doi: 10.1038/s41598-017-17565-x (PMC5727295; doi:10.1038/s41598-017-17565-x)
Supplement: Supplementary file 1 — Supplementary Information [file 41598_2017_17565_MOESM1_ESM.pdf]

Endometrial apoptosis and neutrophil infiltration during menstruation exhibits spatial and temporal dynamics that are recapitulated in a mouse model

Gregory M Armstrong, Jacqueline A Maybin, Alison A Murray, Moira Nicol, Catherine Walker, Philippa TK Saunders, Adriano G Rossi and Hilary OD Critchley

Supplementary Data: Tables (1), Figures (2)

Supplementary Table 1

Immunohistochemistry

Laboratory Code	Cycle Day	Cycle Min./Max. (days)	Histological Stage	Serum [E2] (pM)	Serum [P4] (nM)
AR169	21 (-5)	21/28	Late Secretory	819	6.37
AR172	24 (-5)	28/28	Mid Secretory	1949	94.25
AR103	25 (-4)	28/28	Late Secretory	467	17.6
AR189	26 (-3)	28/28	Late Secretory	176	11.29
AR218	29 (-2)	28/32	Late Secretory	168	4.44
AR180	28 (-1)	28/28	Late Secretory	275	5.3
AR104	33 (-1/0)	30/31	Late Secretory	495	22
AR196	1	-	Late Secretory/Menstrual	392	3.06
AR129	1	-	Menstrual	151	4.31
AR211	2	-	Menstrual	153	1.61
AR214	2	-	Menstrual	176	2.55
CT1185EM	3	-	Menstrual	242	<3
AR212	5	-	Weakly Proliferative	104	2.47
AR161	6	-	Proliferative	318	2.6
AR223	7	-	Late Proliferative	340	6.38
CT892EM	7	-	Proliferative	424	<3
CT2015EM	8	-	Proliferative	607	<3
CT787EM2	8	-	Proliferative	928	<3
CA748EM	8	-	Proliferative	215	<3
AR136	9	-	Mid Proliferative	1002	13.68
CT1215EM	10	-	Proliferative	275	<3
AR118	10	-	Mid Proliferative	311	1.4
CT1148EM	11	-	Proliferative	372	<3
AR125	11	-	Mid Proliferative	842	7.1

RT-qPCR

Laboratory Code	Cycle Day	Cycle Min./Max. (days)	Histological Stage	Serum [E2] (pM)	Serum [P4] (nM)
CA85E	23	-	Late Secretory	90.6	11.51
CH325E	28	-	Late Secretory	427.6	19.84
CH315E	2	-	Menstrual	108	3.16
CT1142E	2	-	Menstrual	129	<3
CT1185E	3	-	Menstrual	242	<3
CH1004E	5	-	Menstrual	140	<3
CT1370E	6	-	Proliferative	260.11	<3
CT1070E	7	-	Proliferative	270	<3
CT1114E	7	-	Proliferative	215	<3
CH996E	8	-	Proliferative	672	<3
CT1384E	11	-	Proliferative	879.31	<3

Patient sample details for ‘full-thickness’ (uterine lumen to myometrial/endometrial junction) and ‘Pipelle’ endometrial biopsies used for immunohistochemical and RT-qPCR studies. Cycle day determined from last menstrual period reported by patient, or by subtracting from the mean of reported cycle minima/maxima (given in parentheses); histological stage determined by pathologist. E2 = oestradiol, nM = nanomolar, pM = picomolar, P4 = progesterone.

Supplementary Figure 1

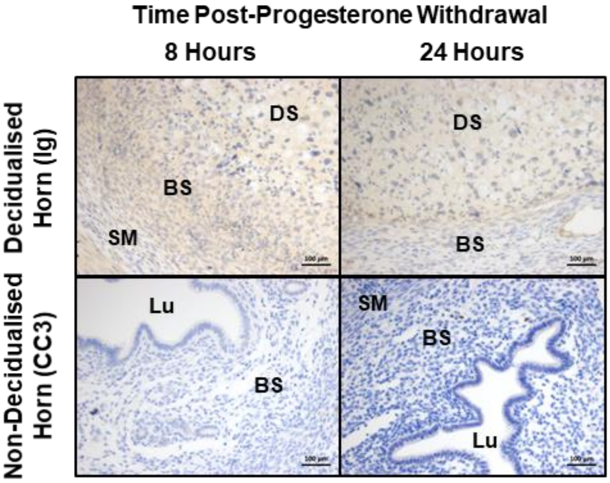

Isotype negative controls (top) show no staining, and non-decidualised tissue controls (bottom) show no CC3 immunoreactivity, confirming "menstrual" apoptotic changes are decidualisation-specific. Negative controls for Fig. 3a (cleaved-caspase-3-immunostained mouse endometrium). Photomicrographs of mouse uterus (decidualised horn) tissue sections from 8h and 24h after progesterone withdrawal, incubated with non-immunised rabbit Ig fraction in place of anti-cleaved caspase-3 antibody (top); and of mouse uterus (non-decidualised horn) tissue sections from 8h and 24h after progesterone withdrawal, incubated with anti-cleaved caspase-3 antibody (bottom).
BS = basal stroma, DS = decidualised stroma, Lu = lumen, SM = smooth muscle.

Supplementary Figure 2

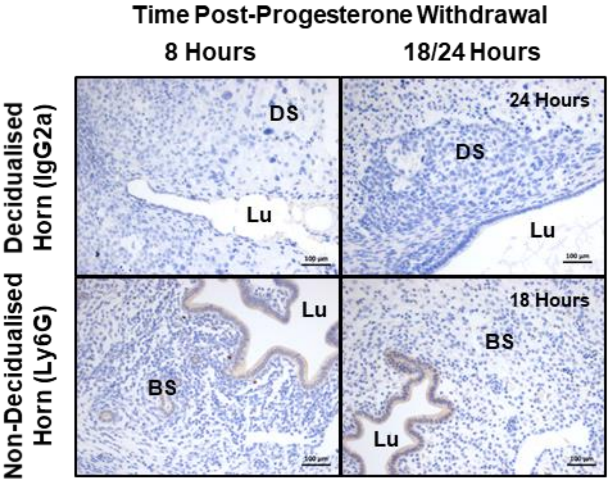

Isotype negative controls (top) show no staining, and non-decidualised tissue controls (bottom) show negligible Ly6G-immunopositive leukocytes, confirming "menstrual" neutrophil infiltration is decidualisation-specific. Negative controls for Fig. 5a (Ly6G-immunostained mouse endometrium). Photomicrographs of mouse uterus (decidualised horn) tissue sections from 8h and 24h after progesterone withdrawal, incubated with rat IgG2a in place of anti-Ly6G antibody (top); and of mouse uterus (non-decidualised horn) tissue sections from 8h and 18h after progesterone withdrawal, incubated with anti-Ly6G antibody (bottom).

BS = basal stroma, DS = decidualised stroma, Lu = lumen.
